# Extracting band edge profiles at semiconductor heterostructures from hard-x-ray core-level photoelectron spectra

**DOI:** 10.1038/s41598-020-69658-9

**Published:** 2020-08-03

**Authors:** Peter V. Sushko, Scott A. Chambers

**Affiliations:** Physical Sciences Division, Physical and Computational Sciences Directorate, Pacific Northwest National Laboratory, Richland, WA 99352 USA

**Keywords:** Characterization and analytical techniques, Computational methods, Electronic and spintronic devices

## Abstract

Internal electric fields that underpin functioning of multi-component materials systems and devices are coupled to structural and compositional inhomogeneities associated with interfaces in these systems. Hard-x-ray photoelectron spectroscopy is a valuable source of information on band-edge profiles, governed by the distribution of internal fields, deep inside semiconductor thin films and heterojunctions. However, extracting this information requires robust and physically meaningful decomposition of spectra into contributions from individual atomic planes. We present an approach that utilizes the physical requirements of a monotonic dependence of the built-in electrostatic potential on depth and continuity of the potential function and its derivatives. These constraints enable efficient extraction of band-edge profiles and allow one to capture details of the electronic structure, including determination of the signs and magnitudes of the band bending as well as the valence band offsets. The utility of this approach to generate quantitative insight into the electronic structure of complex materials is illustrated for epitaxial $\hbox {SrTiO}_3$ on intrinsic Si(001).

## Introduction

The rapidly growing plethora of low-dimensional materials stimulates interest in engineering materials systems that exhibit internal electric fields which, in turn, enable novel functionalities^1^. Thin-films, heterostructures, superlattices, and multi-layers of complex oxides have long been considered a versatile platform for the design of quasi-two-dimensional (2D) materials in which electric fields can be induced by controlled synthesis of asymmetric interfaces^2,3^. Interface-driven properties in such systems find their applications in oxide electronics^4^, ferroelectrics^5^, and catalysis^6^ among others. For example, internal fields enable efficient splitting of electron-hole pairs and the formation of quasi-2D electron and hole gases. One of the well-known examples of such behavior is the formation of 2D electron gas at the $\hbox {LaAlO}_3$/$\hbox {SrTiO}_3$ (LAO/STO) interface^7^, while an observation of a 2D hole gas on the complementary STO/LAO interface was reported only recently^8^. The existence of internal fields is often detected by performing transport measurements. However, these measurements alone do not capture the details of built-in potential profiles, which makes it challenging to quantitatively relate interfacial properties and near-interface defects and disorder. Yet, such insights are critical for the predictive design of functional materials. For example, tunneling measurements on the LAO/STO interface revealed the internal field of 80 meV/Å^9^, which is much lower than the field (240 meV/Å) predicted by ab initio simulations for the digital interface structure^10^, while only negligible field was found using x-ray photoelectron spectroscopy (XPS) measurements^11^. These discrepancies indicate that electronic properties of polar interfaces are sensitive to structural and chemical inhomogeneities and suggest that information about these inhomogeneities can be deduced from the knowledge of the built-in potential profiles^12^.

The band-edge profile in heterostructures is the variation of band-edge energy in the direction normal to the interface. In multi-layer structures, band-edge profiles comprise band bending within each layer and band offsets at the junctions of these layers. For well-understood and well-characterized semiconductor interfaces, such as those consisting of Group III–V semiconductors, potential profiles can be readily calculated using classical Poisson–Boltzmann approach^13^ or the Schrödinger–Poisson method^14^. However, for heterojunctions (HJ) involving complex oxides, the accuracy of such calculations is limited: a lack of detailed information on composition, the types of electrically active defects, and their spatial distribution^15^ introduce uncertainties in the distribution of charge and the associated strengths and directions of electric fields.

The engineering of potential profiles and, accordingly, the design of physical structures that give rise to such profiles, is based on relationships between band offsets, lattice strain, deformation potentials^16–18^, relative positions of bulk band-edges^19^ and the character of interatomic bonds^20^. In spite of progress in this area, including the development of theoretical approaches to determine the innate band alignment between solids^21^, quantitative characterization of the resulting potential profiles remains a challenge. Electronic transport measurements yield important data, such as carrier concentrations, mobilities and surface potentials. However, these measurements do not produce spatially resolved information needed to construct quantitatively accurate band-edge profiles^22^.

A promising route to obtaining band-edge profiles is based on the analysis of core-level x-ray photoelectron spectra, particularly those measured with hard x-rays (HAXPES). Hard x-rays generate photoelectrons with larger attenuation lengths and, accordingly, capture band-edge profiles over longer length scales. This approach relies on the fact that core orbitals are highly spatially localized and their ionization potentials (binding energies) track with depth-dependent changes in the electrostatic potential^23^. At the same time, core-level spectra contain contributions from all atomic planes across the probe depth of the measurement, rendering deconvolution of the measured signal into individual contributions from specific atomic planes a challenging problem.

In superlattices, this problem can be partially addressed using an approach known as standing wave XPS/HAXPES: by sweeping the incident angle at different x-ray energies, it is possible to selectively enhance photoemission signals from different regions of the superlattice^24–28^. However, this approach is not applicable to multilayer structures that lack well-defined periodicity in the off-interface direction.

Here we propose a method that does not require long-range periodicity and is well suited for single heterojunctions. Our method allows band-edge profiles to be extracted from only two core-level spectra, one from each side of a buried interface. We demonstrate this method using a prototypical complex oxide/semiconductor $\hbox {SrTiO}_3$/Si HJ and HAXPES spectra excited with 6 keV x-rays^12^ and discuss physical insight obtained for this system.

**Figure 1:**
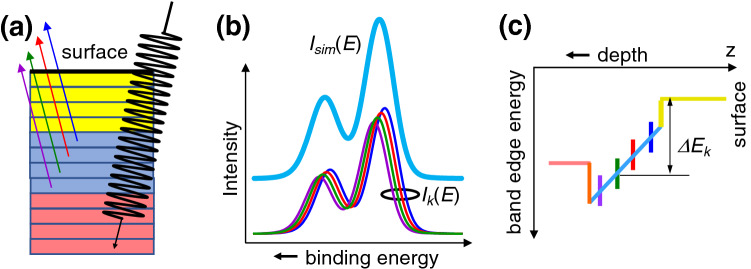
General approach to reconstructing band-edge profiles. (**a**) Core-level photoemission from each atomic plane contributes to the full XPS spectrum. (**b**) The spectrum $I_{k}(E)$ from atomic plane *k* ($k=1,2,\ldots$) is shifted by energy $\Delta E_k$ (**c**) and its intensity is attenuated according to the distance from plane *k* to the surface. The sign of the binding energy shifts $\Delta E_k$ is determined by the gradient of the built-in potential. The simulated spectrum $I_{sim}(E)$ is the sum of spectra $I_{k}(E)$ from all planes. The effect of the built-in potential on the spectrum is an asymmetric broadening. (**c**) The potential profile is reconstructed using $\Delta E_k$ values obtained by fitting the superposition $I_{sim}(E)$ of individual plane contributions $I_{k}(E)$ to the measured spectrum.

**Figure 2:**
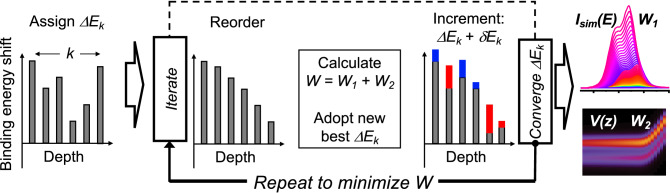
Spectral fitting procedure schematic. Going from left to right, initial trial binding energy shifts $\Delta E_k$ are randomly generated and assigned to each atomic plane *k*. The iteration loop, shown in the central panel, includes (i) ordering $\Delta E_k$ to generate a monotonic function of *k*, (ii) calculation of the cost function *W*, after which the best trial set of $\Delta E_k$ that minimize *W* is adopted, and, (iii) changing $\Delta E_k$ by $\delta E_k$, which can be either positive (blue) or negative (red). This process is repeated until convergence is reached. The optimal set of shifts $\Delta E_k$ simultaneously reproduce the experimental spectrum (top right) and provide a smooth potential profile (bottom right), as quantified by the magnitudes of $W_1$ and $W_2$, respectively.

## Results and discussion

### Reconstructing band-edge profiles

Our approach to extracting the band-edge profile along the vertical direction is illustrated in Fig. 1. It is assumed that each atomic plane *k* ($k=1,2,\ldots$) within the probe depth contributes to the heterojunction spectrum *I*(*E*) and that each contribution $I_k(E)$ has the same lines hape as a reference spectrum $I_0(E)$ of the corresponding bulk material measured for a phase-pure specimen in a flat-band state^12^. We start by shifting the peak channel energy for each spectrum $I_k(E)$ with respect to that of the reference spectrum $I_0(E)$ by $\Delta E_k$. Then, the variations of $\Delta E_k$ with depth correspond to variations of the internal potential profile *V*(*z*) calculated at the location of each atomic plane $z_k$, defined as the distance from plane *k* to the surface plane ($k=1$). Accordingly, constructing the correct profile *V*(*z*) is equivalent to finding the optimal set of energy shifts $\Delta E_k$ within the probe depth.

**Figure 3:**
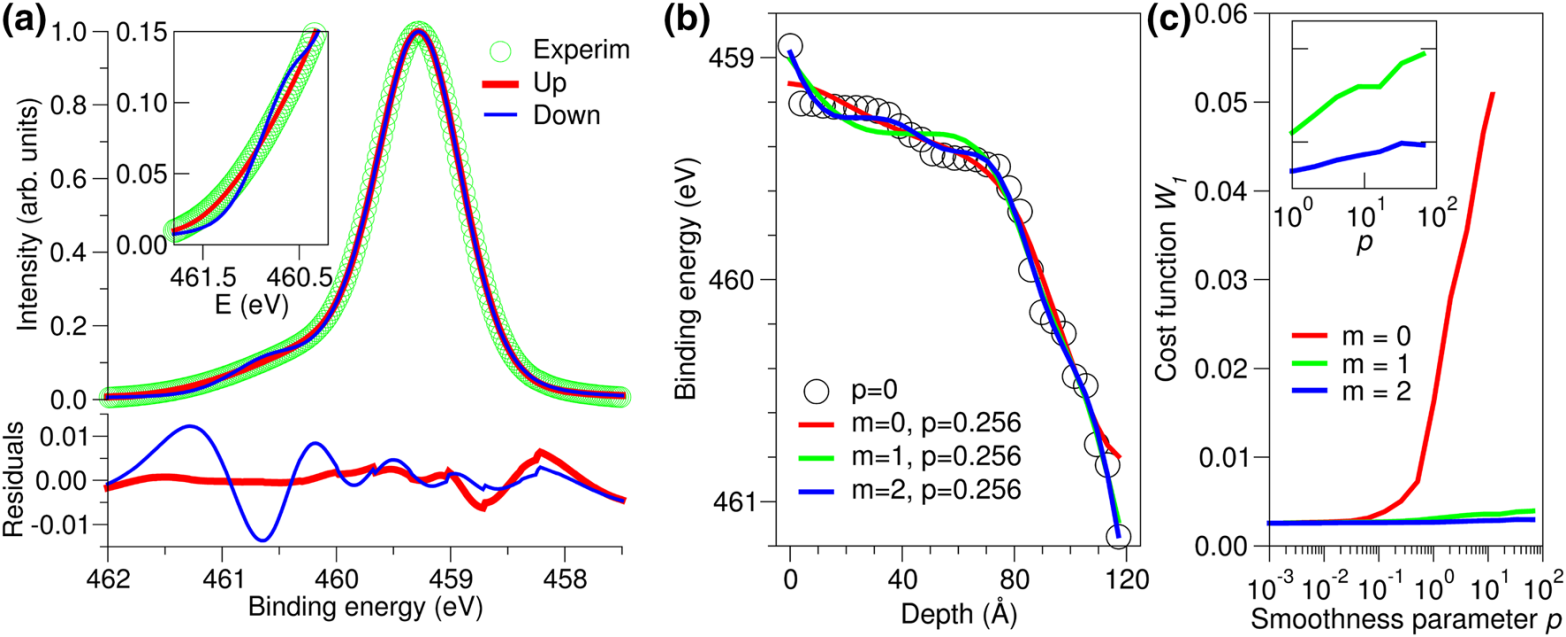
Ti(IV) 2$\hbox {p}_{3/2}$ photoemission in the STO/Si heterojunction. (**a**) Comparison of the simulated and experimental spectra demonstrates that a better fit is obtained for the potential bending up (red) toward the surface than for the potential bending down (blue). The $\sim$ 1.5 eV wide interval (inset) covers the asymmetric shoulder where the fits are sensitive to the sign of the potential gradient. (**b**) Energies corresponding to the peaks of the layer-resolved spectra $I_k(E)$, simulated for with $p=0$ (no imposed smoothness), and with $p=0.001\times 2^{8}$ in conjunction with $m=0,1,2$; see Eq. (4). (**c**) Cost function $W_1$, indicating accuracy of the spectral fitting, shows that for $m=1$ and $m=2$, the simulated and experimental Ti $2\hbox {p}_{3/2}$ spectra agree well over a wide range of *p*. Inset in (**c**) shows that $m=2$ provides insignificant improvement over $m=1$.

**Figure 4:**
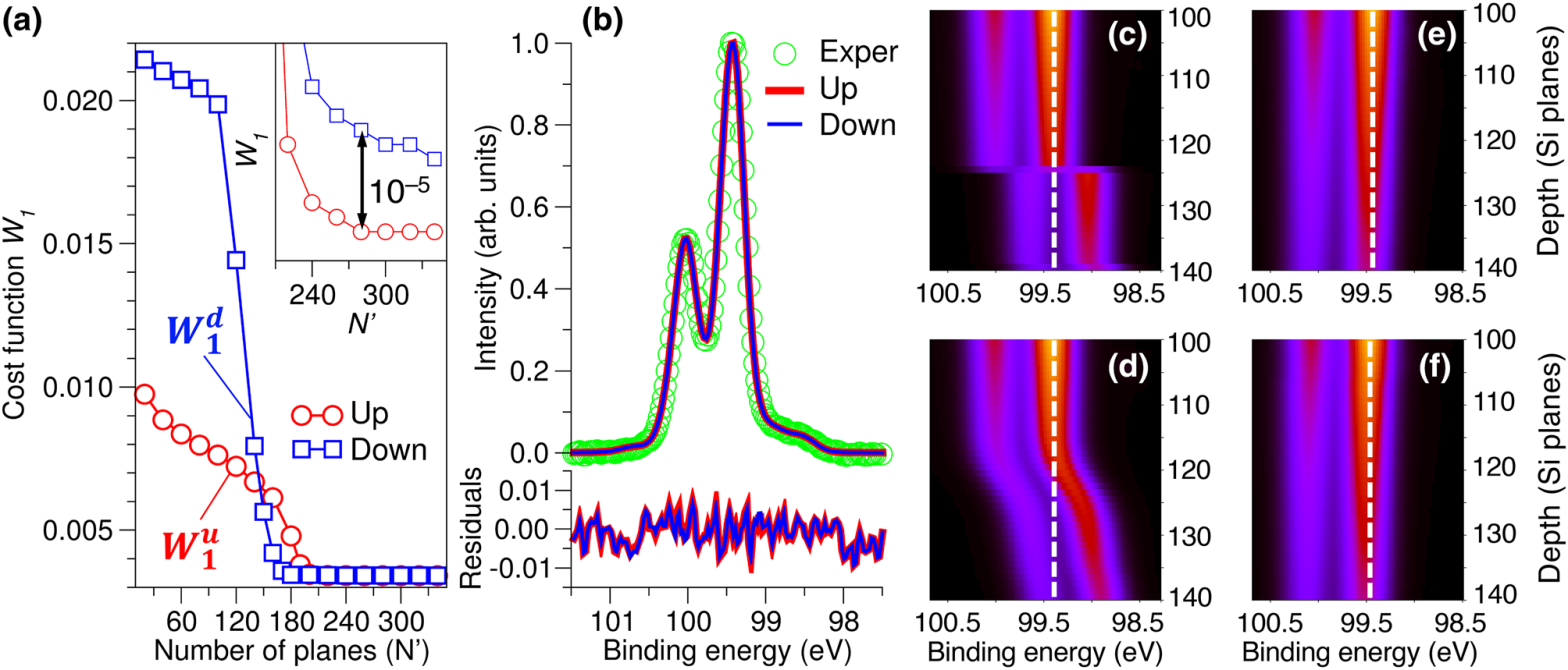
Si 2p photoemission in the STO/Si heterojunction. (**a**) Cost function $W_1$ ($p=0$) indicating the accuracy of fitting the experimental HAXPES spectrum as a function of the number $N'$ of Si planes, for which $\Delta E_k$ values ($k=1,\ldots ,N'$) were explicitly optimized. In all cases, contributions from 350 planes were included. Inset in (**a**): for $N'>$ 240, simulated spectra for the up and down band bending directions agree with the experimental spectrum equally well. (**b**) Comparison of the experimental and simulated Si 2p spectra for the upward (red) and downward (blue) band bending for $N'=340$. (**c**)–(**f**) Color maps of the layer-resolved Si 2p spectra simulated for the down (**c**,**d**) and up (**e**,**f**) band bending directions. Plots in the top row (**c**,**e**) were obtained without smoothness correction ($p=0$) whereas smoothness parameters $m=1$ and $p=0.001\times 2^8$ [see Eq. (4)] were used to generate spectra in the bottom row (**d**,**f**). The sharp knee observed for the case of downward band bending at the depth of $\sim$125 atomic planes is unphysical. No such behavior was found for upward band bending.

The intensity of each contribution $I_k(E)$ is attenuated using an inelastic damping factor of the form $exp(-z_k/\lambda \sin ( \theta ))$, where $\lambda$ is the electron attenuation length and $\theta$ is the take-off angle, defined so that $\theta =90^{\circ }$ corresponds to the direction normal to the surface. Thus, the contribution from plane *k* can be expressed as

1 $$\begin{aligned} I_k(E) = exp\left( -\frac{z_k}{\lambda \sin (\theta )}\right) I_0(E+\Delta E_k) \end{aligned}$$

and the total simulated photoemission spectrum is given by

2 $$\begin{aligned} I_{sim}(E) = \sum _{k=1}^{N} exp\left( -\frac{z_k}{\lambda \sin (\theta )}\right) f_k I_0(E+\Delta E_k). \end{aligned}$$

Here, the upper limit for the summation is determined either by the number of atomic planes *N* (e.g., for films thinner than the probe depth) or by the electron attenuation length. For semi-infinite systems, such as macroscopic substrates, we define the probe depth as the number of planes *N* that cumulatively generate 99.9% of the total signal intensity. Assuming a Beer’s law type dependence and $\theta =90^\circ$, this probe depth amounts to   6.9$\lambda$. The optimal set of $\Delta E_k$ is found iteratively so that $I_{sim}(E)$ fits the experimental spectrum $I_{exp}(E)$. The factors $f_k$ are occupancies of the species of interest in the atomic planes *k*. Unless stated otherwise, we assume that $f_k=1$ for all *k*.

**Figure 5:**
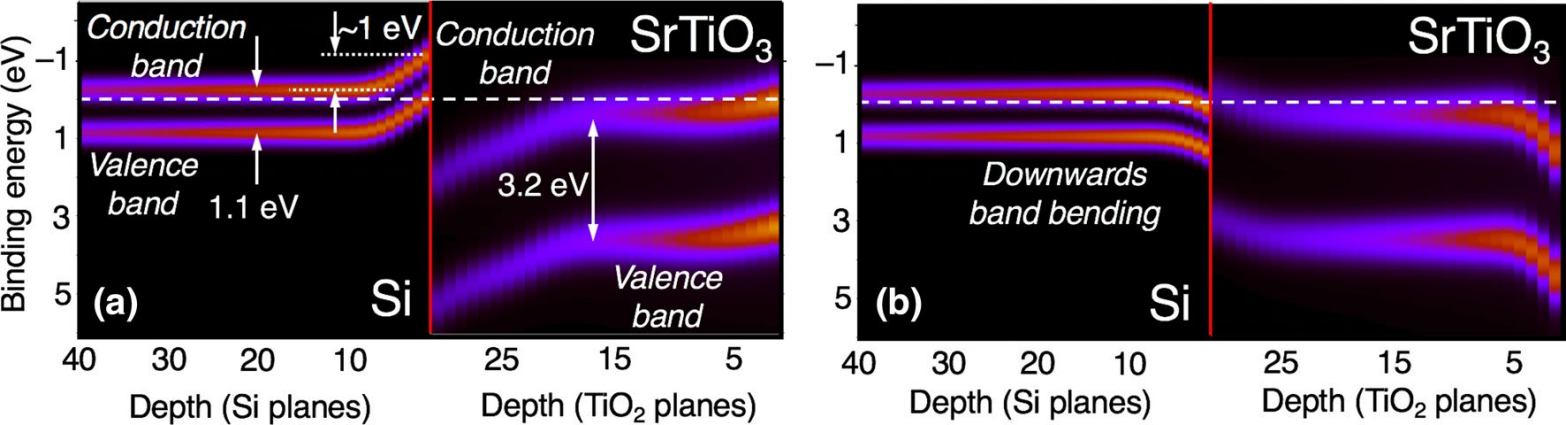
Color maps for the valence band (VB) and conduction band (CB) edge variations with depth in the Si substrate (left) and $\hbox {SrTiO}_3$ film (right), constructed using layer-resolved Si 2p and Ti 2$\hbox {p}_{3/2}$ spectra for the cases of upward (**a**) and downward (**b**) band bending. The distances from the surface (right) and from the $\hbox {SrTiO}_3$/Si interface (left) are indicated in terms of $\hbox {TiO}_2$ and Si atomic planes, respectively. The dashed line corresponds to the Fermi level used to reference HAXPES spectra utilized in this work. The interfaces are shown with the vertical red lines.

**Figure 6:**
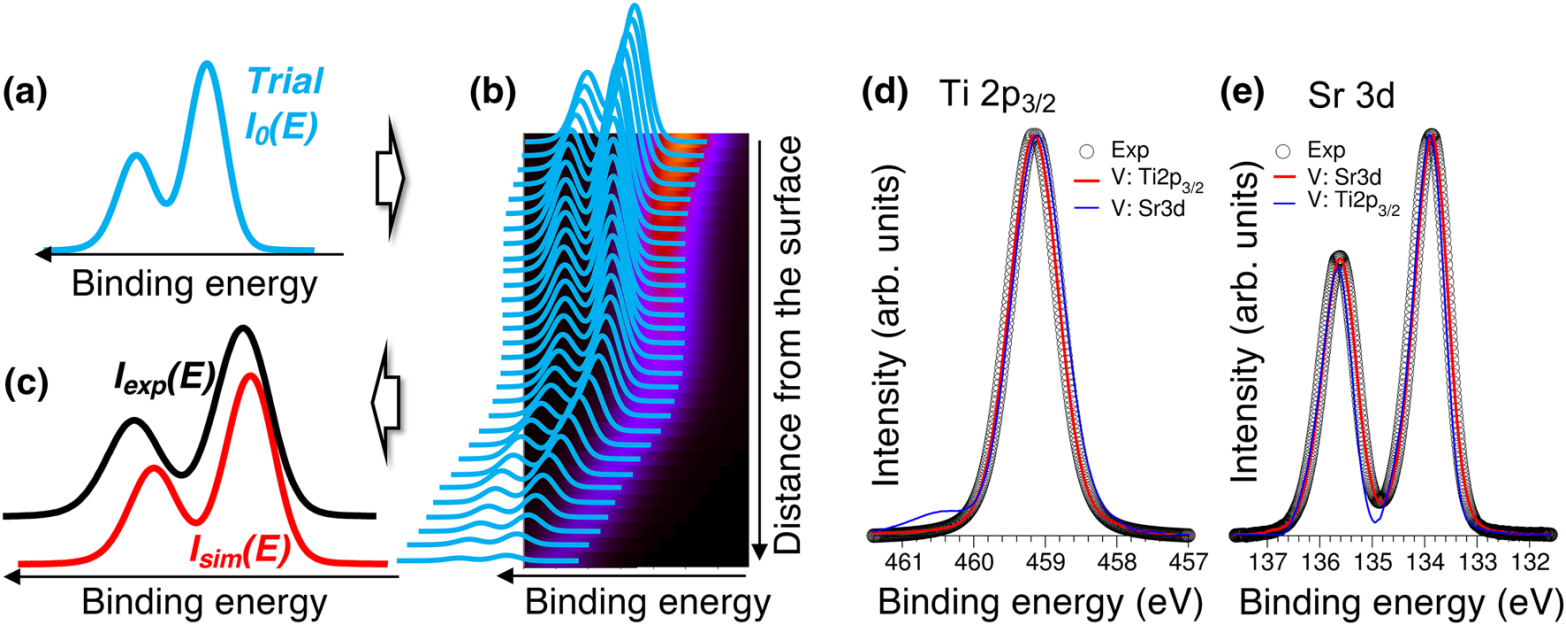
(**a**–**c**) Reconstruction of the basis line shape $I_0(E)$ (**a**) from the known band bending potential profile (**b**) and measured spectrum $I_{exp}(E)$ acquired for a heterojunction (**c**) using the algorithm outlined in Fig. 2. (**d**,**e**) Comparison of the Ti 2$\hbox {p}_{3/2}$ and Sr 3d line shapes measured in the flat-band condition for bulk $\hbox {SrTiO}_3$ along with line shapes reconstructed using potential profiles *V* derived by fitting Ti 2$\hbox {p}_{3/2}$ and Sr 3d spectra for STO/Si HJ.

In previous reports, built-in potential profiles were usually represented by a linear function or a piecewise-function consisting of linear segments^29,30^. While this approach allows one to obtain fits with few tunable parameters, it cannot capture the detailed profile of *V*(*z*) which is essential for in-depth understanding of heterojunction electronic structure. Our algorithm determines $\Delta E_k$ without the linearity constraint. Instead, the explored space of $\Delta E_k$ solutions is constrained by applying two physical criteria: (i) *V*(*z*) is monotonic in *z*, and, (ii) *V*(*z*) is a smooth function, i.e., its derivatives $V'$, $V'',\ldots$ are continuous.

In this approach, we assign an appropriate reference spectrum $I_0(E)$ to each atomic plane within the probe depth of the material^31^. To calculate $I_0(E)$, we generate its analytical representation by fitting the reference spectrum to a linear combination of Gaussian (*G*) and Lorentzian (*L*) functions:

$$\begin{aligned} I_0(E) = \sum _{i=1}^{n} \left[ g_i G(E-E^{G}_i,\sigma _i) + h_i L(E-E^{L}_i,\gamma _i)\right] . \end{aligned}$$

Here

$$\begin{aligned} G(x,\sigma ) = \frac{1}{\sigma \sqrt{2\pi }} exp\left( -\frac{x^2}{2\sigma ^2}\right) , \,\,\,\,\,\,\, L(x,\gamma ) = \frac{1}{\pi } \frac{\gamma }{x^2 + \gamma ^2}, \end{aligned}$$

and $g_i$ and $h_i$ are fitting coefficients; $I_0(E)$ is normalized so that the peak intensity is unity. In the case of the Ti 2$\hbox {p}_{3/2}$ spectrum measured for clean STO(001), one $G(x,\sigma )$ and one $L(x,\gamma )$ function yield a fit with the root mean square deviation (RMSD) of less than 0.004. In the case of the Si 2p spectrum for Si(001), two $G(x,\sigma )$ and two $L(x,\gamma )$ functions fit the experimental spectrum with an RMSD of less than 0.002. The experimental spectra, best fits, and corresponding residuals for both Ti 2$\hbox {p}_{3/2}$ and Si 2p are shown in Fig. S1.

The algorithm is shown schematically in Fig. 2. The fitting starts by assigning randomly generated binding energy shifts $\Delta E_k$ ($k=1,\ldots ,N$) to the *N* planes starting from the plane closest to the surface ($k=1$). These energy shifts are sorted in either increasing or decreasing order, depending on the assumed sign of $\partial V/\partial z$, and are then reassigned to the lattice planes, yielding a potential that changes monotonically with depth. Both potential gradient signs are modeled independently in searching for the best fit.

Following this assignment, spectral contributions from all planes are summed to generate a trial simulated spectrum, $I_{sim}(E)$, according to Eq. (2). The $I_{sim}(E)$ and $I_{exp}(E)$ spectra are compared by calculating the cost function *W*. Next, a new trial set of $\Delta E_k$ is generated by subjecting the energy shifts to incremental changes (i.e. $\Delta E_k \rightarrow \Delta E_k + \delta E_k$) for which both the magnitude and sign of $\delta E_k$ are selected at random. The new values of $\Delta E_k$ are then again reordered and reassigned to ensure that *V*(*z*) remains a monotonic function. The process is repeated until values of $\Delta E_k$ that result in the best agreement between the measured and simulated heterojunction spectra are found (see Fig. 2).

The cost function *W* consists of two terms, $W_1$ and $W_2$, where

3 $$\begin{aligned} W_1 = \sqrt{\frac{1}{M}\sum _{i=1}^{M} \left[ I_{exp}(E_i) - I_{sim}(E_i)\right] ^2 } \end{aligned}$$

and

4 $$\begin{aligned} W_2(m,p) = p \sum _{k=1}^{N-1} w_k \left[ \varepsilon _{max}^{m}(k) - \varepsilon _{max}^{m}(k+1) \right] ^2, \qquad \text {where} \qquad w_k = exp\left( -\frac{z_k}{\lambda \sin (\theta )}\right) . \end{aligned}$$

Here $W_1$ quantifies the goodness of the fit between the experimental spectrum measured at binding energies $E_i$ ($i=1,2,\ldots ,M$) and the simulated spectrum calculated at the same energies, and $W_2$ is designed to ensure continuity and smoothness of the potential function *V*(*z*); $\varepsilon _{max}^{0}(k)$ is the energy corresponding to the maximum of spectrum $I_k(E)$ for plane *k*. The weighting factor *p* ($p \ge$0) is included to scale the influence of the potential smoothness condition relative to that of the spectral fit. Since the intensities $I_k(E)$ and their contributions to $W_1$ decrease exponentially with *k*, we introduce attenuation factors $w_k$ to impose exponentially decreasing contributions to $W_2$ as well.

To explain the dependence on *m*, we note that for a given potential drop across the probe depth $\Delta V = \left| \varepsilon _{max}^{0}(1)-\varepsilon _{max}^{0}(N) \right|$, term $W_2(0,p)$ reaches its minimum if all values $\delta V = |\varepsilon _{max}^{0}(k) - \varepsilon _{max}^{0}(k+1)|$ for $k=1,2,\ldots$ are equal, i.e., the potential *V*(*z*) is a linear function of *z*. This linearity condition is never satisfied because $W_2$ is optimized not in isolation but together with $W_1$. However, if the relative weights of $W_1$ and $W_2$ are significantly different, the fitting procedure can skew the outcome to give rise to either discontinuities in *V*(*z*) (for $p \approx 0$) or a forced near-linear form of *V*(*z*) (for $p \gg 1$), both of which are unphysical.

To avoid such unphysical solutions, we define first differences between the values of the peak binding energies ($m=1$):

$$\begin{aligned} \varepsilon _{max}^{1}(k) = \varepsilon _{max}^{0}(k) - \varepsilon _{max}^{0}(k+1) \end{aligned}$$

and note that $W_2(1,p)$ reaches its minimum if $\varepsilon _{max}^{1}(k)$ is a linear function of *k*. This would correspond to a quadratic form of $\varepsilon _{max}^{0}(k)$, and therefore, a quadratic dependence of *V*(*z*) on depth. Likewise, second ($m=2$) differences

$$\begin{aligned} \varepsilon _{max}^{2}(k) = \varepsilon _{max}^{1}(k) - \varepsilon _{max}^{1}(k+1) \end{aligned}$$

cause $W_2(2,p)$ to reach its minimum if $\varepsilon _{max}^{0}(k)$ and, thus, *V*(*z*) are cubic functions of depth.

As we discuss below, $W_1$ and $W_2$ are coupled. That is, increasing the value of parameter *p* leads to a smoother potential profile but a poorer fit of $I_{sim}(E)$ to $I_{exp}$(E). For the cases considered here, $m=1$ and $m=2$ provide similar solutions over a wide interval of *p*, suggesting that using values of $m>$ 2 is unnecessary.

### Determination of the built-in potential at the epitaxial $\hbox {SrTiO}_3$/Si(001) heterojunction

We demonstrate the use of this algorithm with the example of epitaxial $\hbox {SrTiO}_3$ film grown on the (001) surface of intrinsic, i.e., undoped Si (*i*-Si)^12^. The film thickness is $\sim$12 nm, which corresponds to 31 $\hbox {TiO}_2$ planes. Here and below we used $\lambda _{\text {Ti}}=60$ Å and used $\theta =85^\circ$ take-off angle unless stated otherwise^31^. We first neglected the $W_2$ term by setting *p* to zero and fitted the Ti(IV) 2$\hbox {p}_{3/2}$ experimental spectrum for the positive and negative gradients of the built-in potential. The two simulated spectra are compared to each other and to the experimental spectrum in Fig. 3a. While the asymmetric line shape is well reproduced in both cases, the numerical agreement between the simulated and the experimental spectra is markedly better when the potential decreases from the interface to the surface (bands bend upward toward the surface) than for the opposite gradient sign (bands bend downward). The root-mean square deviations between the simulated and the experimental spectra ($W_1$) are 0.0026 and 0.0053 for these two cases, respectively (Fig. 3a).

Since the peak binding energies $\varepsilon _{max}^{0}(k)$ scale linearly with the local electrostatic potential, the dependence of $\varepsilon _{max}^{0}(k)$ on the plane number *k* is equivalent to, but offset from, the dependence of *V*(*z*) on *z*. Several profiles of the layer-resolved $\varepsilon _{max}^{0}(k)$ obtained by fitting the Ti 2$\hbox {p}_{3/2}$ spectrum are shown in Fig. 3b for the model with upward band bending. If fitting is done neglecting the smoothness condition ($p=0$), the resulting potential profile has discontinuities in its first derivative that are clearly visible for $z \approx 0$ Å and in the interval 80–120 Å. These discontinuities can be eliminated using $p>$ 0, as also seen in Fig. 3b for $p=$ 0.256 and a range of *m* values. However, at large values of *p*, the potential smoothness term $W_2$ dominates the total cost function, leading to a poor agreement between the experimental and simulated spectra.

In order to evaluate the effect of the smoothness parameters on the quality of the fit, we optimized the total cost function *W* for selected *p* and *m* and plotted the resulting values of $W_1$, as given by Eq. (3) in Fig. 3c. If $m=0$ and $p\ge$ 0.064, the discontinuities in the potential profile are eliminated, as shown in Fig. 3b for $p=0.256$. However, this comes at the price of $W_1$ rapidly increasing with *p*, resulting in the loss of agreement between $I_{sim}$(Ti) and $I_{exp}$(Ti), as manifested by the escalation of $W_1$ in Fig. 3c. In contrast, for $m=1$ and $m = 2$, $W_1$ remains nearly constant over a wide range of *p*, i.e., $I_{sim}$(Ti) is a good fit to $I_{exp}$(Ti) and, simultaneously, the resulting potential is a smooth function of depth, as shown in Fig. 3b.

The situation is more complex in the case of the Si 2p spectrum measured for the same STO/Si heterojunction. Here, the HAXPES spectrum includes contributions from a much larger number of atomic planes. We truncate the summation in Eq. (2) at the depth that provides 99.9% of the total intensity and rescale the resulting spectrum to match the peak intensity of the experimental spectrum. For the bulk Si lattice constant of 5.43 Å and an attenuation length of 70 Å^32^, this depth corresponds to $N=350$ Si planes.

Optimizing $\Delta E_k$ for all 350 planes is a daunting and unnecessary task. Instead, we investigate whether a good fit to $I_{exp}$(Si) can be obtained by optimizing $\Delta E_k$ for $N'$ ($N' < N$) planes. In this approach, the contributions of the remaining $N-N'$ planes are also taken into account but their binding energy shifts are equal to $\Delta E_{N'}$.

The cost function $W_1$ in Fig. 4a shows how the accuracy of the $I_{sim}$(Si) fit to the experimental spectrum depends on $N'$ for $p=0$. Increasing $N'$ corresponds to increasing the number of variable parameters $\Delta E_k$, which, in turn, provides greater variational freedom and results in a lower $W_1$ for both up ($W^u_1$) and down ($W^d_1$) directions of the band bending. For $N'>$ 190, $W^u_1$ and $W^d_1$ are within 10$^{-5}$ of each other. This difference is too small to establish which band bending direction better reproduces the experimental spectrum. Indeed, a direct comparison of $I_{sim}$(Si) for up and down band bending to $I_{exp}$(Si) in Fig. 4b shows that these simulated spectra are nearly identical.

A natural way to differentiate between the fits for the up and down band bending is to examine the dependencies of $W^u_1$ and $W^d_1$ on the smoothness parameters *p* and *m*. In particular, we expect that a set of $\Delta E_k$ that gives rise to a smooth potential *V*(*z*) and, simultaneously, generates a $I_{sim}$(Si) that agrees well with $I_{exp}$(Si) will determine the sign of the band bending. To illustrate the effect of *p* and *m* on the smoothness of *V*(*z*) and on the magnitude of $W_1$, we consider a range of *p* (2$^7 \le$ 1000 $p \le$ 2$^9$) and $m=0,1,2$ and calculate $W_1$ as a function of $N'$ for the upward and downward (toward the surface) band bending directions. The resulting dependencies of $W^u_1$ and $W^d_1$ on $N'$ are shown in Fig. S2.

The behavior of $W^u_1$ for $m=0$ and $p=0.256$ and 0.512, shown in the top panel of Fig. S2(a), is consistent with what was found for the case of Ti 2$\hbox {p}_{3/2}$ (Fig. 3c): $m=0$ and large *p* enforce a smooth potential profile but introduce a significant mismatch between $I_{exp}$ and $I_{sim}$, manifested by the large values of $W^u_1$ for all considered $N'$. In contrast, cost function $W^d_1$ calculated for the same set of *m* and *p* [top panel in Fig. S2(b)] improves with increasing $N'$ suggesting a better fit for the downwards direction of the band bending. However, we find that for all other considered *m* and *p*, $W^u_1$ and $W^d_1$ in Fig. S2 demonstrate similar trends with $N'$ as in the case of $p=0$, shown in Fig. 4a and are, therefore, similarly inconclusive. In particular, since $W_1^u$ can be larger or smaller than $W_1^d$ depending on the specific choice of $N'$, *m* and *p*, these trends do not allow us to unambiguously assign the direction of the band bending.

Since the cost function *W* is constructed to be sensitive to (1) the goodness of $I_{sim}(E)$ fit to the experimental spectrum $I_{exp}(E)$ *and* (2) the smoothness of the binding energy profile simultaneously, important details of the electronic structure can be skewed by the competition between these two drivers. Hence, relying on minimization of *W* alone may not be sufficient to obtain the correct potential profile. One can obtain additional insight into the direction of the band bending by analyzing the layer-resolved spectra $I_k(E)$ directly. Color contour maps in Fig. 4c–f show the intensity of the best-fit families of Si 2p spectra as a function of the depth for the downward and upward band-bending directions. Here we focus on the depth interval of 160–175 Å from the STO/Si interface, which corresponds to the interval between 120th and 130th Si lattice planes.

In the case of downward bending (Fig. 4c,d), there is a large binding energy shift at $\sim$ 167 Å (plane 124). This shift is present for all considered *p* and *m* that provide satisfactory agreement between $I_{sim}$(Si) and $I_{exp}$(Si) and it occurs at the same region of $N'$ where the cost function term $W^d_1$ (see Fig. 4a) decreases sharply from $\sim$ 0.2 to $\sim$ 0.035. In other words, Si 2p binding energy discontinuity is necessary to reproduce the experimental HAXPES spectrum. While it is known that internal interfaces often result in band offsets which can manifest themselves as discontinuities of potential *V*(*z*), there is neither evidence for nor expectations that such discontinuities exist in the Si substrate at the distance of $\sim$ 170 Å from the STO/Si interface. In contrast, in the case of upward bending, the profiles of $I_k$(Si) deep in the Si substrate are nearly independent on the plane number *k* and they are not affected by the smoothness parameters *p* and *m* (Fig. 4e,f). Therefore, we conclude that the good fit obtained for the downward band bending is fortuitous and it is only achievable by introducing artificial discontinuity in the binding energy profile deep in the Si substrate. In contrast, the band bending profile obtained for the upward bending case is consistent with asymptotic convergence of the potential with the distance from the interface.

### Band offsets at the STO/Si(001) heterojunction

Having established that the Ti 2p and Si 2p binding energy profiles with upward band bending near the surface yield high-quality and physically-reasonable fits of the corresponding experimental spectra, we now consider other aspects of the STO/Si heterojunction electronic structure. Continuity of the electric displacement across the interface places restrictions on the potential gradients in the two materials: the relationship

5 $$\begin{aligned} \epsilon _{Si} \left( \frac{\partial V}{\partial z} \right) _{Si} = \epsilon _{STO} \left( \frac{\partial V}{\partial z} \right) _{STO} \end{aligned}$$

links gradients of the potentials on each side of the interface and the corresponding static dielectric constants $\epsilon$. The slopes of the potential ($\partial V(z)/\partial z$) near the interface were determined by linear fitting of the $I_k(E)$ peak energies ($\varepsilon _{max}$) in the three planes on each side of the interface (Fig. S3). These fits reveal that the slopes for Ti 2$\hbox {p}_{3/2}$ and Si 2p are 0.50 and 1.29 eV/nm, respectively. Assuming the dielectric constant of Si ($\epsilon _{Si}$) is 11.7, we estimate the dielectric constant of the STO film to be $\epsilon _{STO} \approx$ 30. This value is consistent with the range of the dielectric constants proposed for MBE-grown thin STO films on Si(001)^33^.

We can now use these data to construct valence band (VB) and conduction band (CB) edge profiles for the heterojunction region. Experimental measurements show that the top of the VB in bulk STO is 455.76 eV above the Ti 2$\hbox {p}_{3/2}$ peak energy^12^. Likewise, the VB edge in bulk Si is measured to be 98.54 eV above the Si 2$\hbox {p}_{3/2}$ peak energy. Subtracting these values from the associated binding energy profiles yields the VB edge profiles within the STO and Si. Moreover, subtracting the bulk band gaps (3.25 eV for STO^34^ and 1.12 eV for Si) from the VB band-edge profiles yields the CB band-edge profiles. The resulting band alignments are shown in Fig. 5a. We note that a similar VB band-edge profile (Fig. S4) was obtained by fitting the Sr 3d HJ spectrum and shifting the resulting core-level energy profile by the energy difference between Sr 3$\hbox {d}_{5/2}$ peak energy and VB edge in the bulk STO (130.26 eV).

To corroborate these results, we note that an electronically significant concentration of in-diffused oxygen impurities was detected in Si by secondary ion mass spectrometry (SIMS)^12^. The band alignment shown in Fig. 5a suggests that itinerant electrons in the Si originating from these oxygen donors would drift across the interface into the STO conduction band. This charge transfer bends the Si bands upward to a sufficiently large extent ($\sim$ 1 eV) that a hole gas forms on the Si side of the interface, which is consistent with experimental transport measurements reported elsewhere^12^. Continuity of the electric displacement across the interface requires that the STO bands also bend upward away from the interface.

The relatively short ($\sim$ 10 atomic planes) depletion width in the Si substrate, as seen in Fig. 5a, suggests that Si in the vicinity of the STO/Si interface is no longer intrinsic but, instead, contains a high concentration of charge carriers. Secondary ion mass spectrometry analysis of this region shows significantly higher concentration of oxygen than what is typically found in Czochralski-grown *i*-Si^12^. We propose that this oxygen diffuses into the Si during the epitaxial growth of STO and acts as a shallow donor. The donated electrons drift into the STO, leaving behind a relatively high concentration of ionized donors, and result in a small depletion width^22^.

The band-edge profiles extracted from the HAXPES data (Fig. 5a) can be validated against transport measurements for this system^12^. In particular, the measurements indicate the presence of a dead layer in the STO for temperatures below 300 K, which consistent with surface depletion. The measurements also point to the presence of a hole gas in the Si at temperatures above 300 K, which is consistent with the large, sharp upward band bending on the Si side of the interface. In addition, the magnitude of the band bending extracted from the Hall-effect data (0.9 eV) is in good agreement with that determined from our fitting (0.98 eV).

It is instructive to consider a hypothetical scenario in which the $I_{exp}$(Ti) and $I_{exp}$(Si) fitting is performed less accurately and examine the resulting physical picture. Neglecting the deviation between the simulated and experimental spectra for Ti 2$\hbox {p}_{3/2}$ (inset in Fig. 3a), and disregarding the discontinuity in the Si 2p profile at 123–125 atomic planes from the interface (Fig. 4c,d), may lead to a conclusion that band bending is downward in both materials. The band-edge profiles calculated for this scenario are shown in Fig. 5b. Interestingly, the STO dielectric constant extracted from Eq. (5) for this case ($\sim$ 34) is reasonable. However, it is clear that this band diagram represents a very different electronic structure from that shown in Fig. 5a. The bands bend sharply downward at both the Si side of the interface and the STO surface, suggesting that these regions might support two-dimensional electron gas (2DEG) states. If this were the case, the Hall-effect would be dominated by a high-mobility electron gas associated with the Si, rather than the hole gas that was observed in transport measurements^12^. In other words, the band-edge profiles that do not satisfy our criteria for the highest quality of HAXPES fitting are also inconsistent with the results of the transport measurements.

The band-edge profiles obtained near normal emission can be cross checked using spectra obtained at lower take-off angles ($\theta$). To assess the effect of $\theta$ on band edge profiles, we simulated Ti 2$\hbox {p}_{3/2}$ line shapes for several values of $\theta$ and then fitted them, as described in the SI (see also Figures S5 and S6). As $\theta$ decreases, contributions due to the near-surface planes become more influential in keeping with the shorter probe depths. In our simulations, the sets of optimal $\Delta E_k$ values obtained for all $\theta$ are in near-perfect mutual agreement within their respective probe depths. That said, if $\theta$ is sufficiently small to resolve additional effects associated with surfaces, it will affect the HAXPES line shape and, in turn, affect the potential profile in that region. Thus, performing measurements over a range of take-off angles in order to capture variation of the potential at increasing depth into the sample and resolve surface features is a useful reproducibility check.

The algorithm outlined in Fig. 2 can be utilized in reverse in order to reconstruct the basis spectrum $I_0(E)$ assuming that the potential profile *V*(*z*) and the spectrum for a heterojunction $I_{exp}(E)$ are known. In this case, a trial basis spectrum is generated by randomly assigning intensities on an energy grid with a preselected resolution (Fig. 6a). This trial spectrum is replicated for each atomic plane contributing to the HJ spectrum and shifted along the binding energy scale according to the potential profile *V*(*z*). The intensities of the contributions from each plane are attenuated according to the associated distance from the surface, and their sum is compared to the experimental HJ spectrum $I_{exp}(E)$ (Fig. 6b,c). Then, the trial basis is modified and the process is repeated until convergence is reached.

This procedure was used to reconstruct Ti 2$\hbox {p}_{3/2}$ and Sr 3d basis line shapes. Here, we used potential profiles fitted independently for the Ti 2$\hbox {p}_{3/2}$ and Sr 3d spectra for the STO/Si HJ, without the smoothness correction, i.e., $W_2=0$ (see Fig. S4). The intensity increment was 0.01% of the maximum spectrum intensity and the energy resolution was 0.01 eV. The fitted Ti 2$\hbox {p}_{3/2}$ basis line shape (Fig. 6d) is in a good agreement with the experimental spectrum if the fitting is based on the potential *V*(*z*) derived from fitting the Ti 2$\hbox {p}_{3/2}$ HJ spectrum. In contrast, fitting the Ti 2$\hbox {p}_{3/2}$ HJ spectrum using the potential derived from the Sr 3d HJ spectrum results in an artificial peak at high binding energy. Similarly, extracting the Sr 3d basis line shape (Fig. 6e) from the potential derived from Sr 3d HJ spectrum produces an excellent match to the experimental basis spectrum. However, determining the Sr 3d basis spectrum using the potential derived from the Ti 2$\hbox {p}_{3/2}$ HJ spectrum results in the valley between the two spin-orbit components being too deep. These discrepancies notwithstanding, the overall shapes, widths, and peak ratios obtained for these basis line shapes are in reasonable agreement with experiment. This procedure can be useful for systems where the reference line shapes are poorly defined due to, for example, overlap of several spectroscopic features, surface contamination, or charging, each of which precludes core-level measurements in the flat-band regime.

Finally, we examine whether additional insights can be obtained by strengthening or relaxing the fitting conditions. Comparison of the Si 2p fits for the downward and upward band bending (Fig. 4) suggests that fitting a specific spectrum $I_{exp}(E)$ by minimizing $W=W_1 + W_2$ does not have a unique solution *V*(*z*), and that physically meaningful solutions need to be identified by examining their consistency across the entire HJ. One way to approach this issue is to impose more stringent requirements on continuity of the potential profiles by replacing exponentially decreasing attenuation factors $w_k$ in Eq. 4 with $w_k=1$ for all *k*. In this case, even if the contribution $I_{k}(E)$ from plane *k* to the total spectrum $I_{sim}(E)$ is decreasing exponentially with the distance from the surface, the magnitude of its contribution to the continuity cost function $W_2$ is independent of depth. The effect of this modification is evident from the comparison of the fits for the downwards and upwards band bending in Fig. S7: minimization of the discontinuities in the potential profile for the downward bending (see Fig. 4c,d) results in a larger values of $W_1$, i.e., a less accurate fit to the measured spectrum. This effect enables discrimination between the downward and upwards band bending profiles.

In our approach so far, the number of atomic planes *N* contributing to the Ti 2$\hbox {p}_{3/2}$ spectrum was fixed assuming an ideal epitaxial STO/Si interface. However, in the absence of epitaxial growth or in the case of intermixing across the interface, the effective thickness *N* may not be well defined. To consider this situation, we relaxed the fitting conditions for Ti 2$\hbox {p}_{3/2}$ by assuming that the number of $\hbox {TiO}_2$ planes is not known. The dependence of the $W_1$ cost function on *N* for the STO(12 nm)/Si HJ is shown in Fig. S8(a). The thickness of 12 nm corresponds to 31 $\hbox {TiO}_2$ planes. As *N* is varied from $N=17$ to $N=31$, $W_1$ decreases exponentially, indicating that each atomic plane measurably contributes to the overall spectrum. In contrast, at $N>31$, the magnitude of $W_1$ changes linearly. Comparison of the corresponding band bending profiles (Fig. S8(b)) suggests that their key features are nearly independent on *N* between $N=$27 and $N=$35 and that increasing *N* beyond the physically meaningful STO film thickness of 31 $\hbox {TiO}_2$ planes results in a wider flat band region inside the film, where contributions of individual planes to the Ti 2$\hbox {p}_{3/2}$ spectrum are linearly dependent. Importantly, the width of the depletion regions near the STO surface and STO/Si interface as well as potential drop across the film are largely unaffected.

## Summary

We developed an algorithm for the detailed analysis of HAXPES data that allows one to decompose measured heterojunction core-level spectra into contributions associated with individual atomic planes and extract accurate band-edge profiles. While the algorithm utilizes as many variable parameters as there are atomic layers containing the photo-emitting atoms, the range of acceptable values for each of these parameters is constrained by the condition of monotonic behavior of the built-in potential, as well as by conditions of continuity of the potential and its derivatives. These physically meaningful constraints allow for an efficient spectral fitting procedure, even if the probe depth includes several hundred lattice planes.

The utility of this algorithm to reveal the detailed *z*-dependence of the electric potential is demonstrated for 12 nm $\hbox {SrTiO}_3$/Si (001). The results of this analysis are fully consistent with transport measurements conducted independently^12^. We also demonstrate that not carefully scrutinizing the quality of the spectral fit, e.g., dismissing discontinuities in the band-edge profile far from the interface, can lead to the wrong conclusion about the sign of the potential gradients across the heterojunction and incorrect, even if internally consistent, prediction of the electronic structure.

Detailed band-edge profiles of the kind extracted from HAXPES data using the algorithm presented here cannot be directly determined with this level of detail by any other means at present. Therefore, XPS and HAXPES, correctly interpreted, are highly complementary to transport methods to gain deep understanding of the electronic structure heterostructures for a variety of semiconductors. While we have illustrated the power of this method for the prototypical complex oxide/Group IV semiconductor interface STO/Si(001), there are no constraints that would limit applying this approach to any other class of semiconductor heterojunction.

Finally, we note that the ability to map band-edge profiles is especially important if there is no *a priori* knowledge of the direction that electronic charge will flow upon junction formation. This is the situation for heterostructures involving materials such as complex oxides because complex oxides can contain electrically active defects that trap charge and generate internal electric fields not anticipated by the design of the material system^35^. The formation of such defects can be driven by non-equilibrium synthesis conditions which, in turn, underscores the challenges of predicting band bending and band offsets on the basis of equilibrium theoretical models alone.

### Post-acceptance note

During preparation of this manuscript for publication we became aware of a relevant study^36^.

## Electronic supplementary material

Supplementary Information.
