# Supplementary material for: Extracting band edge profiles at semiconductor heterostructures from hard-x-ray core-level photoelectron spectra
Source: Sci Rep. 2020 Aug 3;10:13028. doi: 10.1038/s41598-020-69658-9 (PMC7400555; doi:10.1038/s41598-020-69658-9)
Supplement: Supplementary file 1 — Supplementary Information. [file 41598_2020_69658_MOESM1_ESM.docx]

**Supplementary information**

**TITLE:** Extracting band edge profiles at semiconductor heterostructures from hard-x-ray core-level photoelectron spectra

**AUTHOR NAMES:** Peter V. Sushko^1^, Scott A. Chambers^1^

**AUTHOR ADDRESS:**

^1^Physical Sciences Division, Physical & Computational Sciences Directorate, Pacific Northwest National Laboratory, Richland, Washington 99352, USA

Corresponding authors’ E-mail address: peter.sushko@pnnl.gov, sa.chambers@pnnl.gov

| ** |
| --- |
| **Figure S1.** Experimental HAXPES Ti 2p_3/2_ (top left) and Si 2p (top right) spectra obtained at the flat-band conditions for clean SrTiO_3_ and intrinsic Si, respectively (circles). Both samples were terminated with (001) surfaces. The corresponding fits using Gaussian (G) and Lorentzian (L) functions (top) and the residuals (bottom) are shown using solid lines. One G and one L were used for Ti 2p_3/2_; two G and two L were used for Si 2p. The resulting root mean square deviations are less than 0.004 for Ti 2p_3/2_ and less than 0.002 for Si 2p. See Ref. [12] in the main text for experimental details. |
| ** |
| **Figure S2.** Accuracy of the I_exp_(Si) spectral fit, given by cost function term W_1_ (see equation (3) in the main text). Here, N' is the number of atomic planes for which ΔE_k_ values were explicitly optimized and p and m are smoothness parameters. Panels (a) and (b) show the quality of the fit, represented by the cost function W_1_, for the two assumed signs of the potential gradient corresponding to upward (W^u^_1_) and downward (W^d^_1_) band bending in the direction from the bulk to the surface. The same set of parameters p was used in all cases: p×1000 = 2^7^, 2^8^, and 2^9^. |

| ** |
| --- |
| **Figure S3.** Color maps of the simulated layer-resolved Si 2p (a) and Ti 2p_3/2_ (b) spectra near the SrTiO_3_/Si interface for the upward band bending scenario. The interface is marked with a vertical red line. The distances (top axis) from the surface (b) and from the SrTiO_3_/Si interface (a) are indicated in terms of TiO_2_ and Si atomic planes, respectively. White lines on both sides of the interface highlight the slope of the electrostatic potential ∂V/∂z, where the z-direction is perpendicular to the plane of the interface. |
| **Consistency between the potential profiles obtained using Ti 2p_3/2_ and Sr 3d spectra.**  The depth profile of the valence band (VB) maximum in the STO film obtained by fitting the Ti 2p_3/2_ spectrum (Figures 5 and S3) was independently validated by performing a similar fitting procedure for the Sr 3d spectrum. In both cases we used spectra collected for the same STO (12 nm) / Si heterojunction and assumed the same thickness of 31 atomic planes. Here we used electron attenuation lengths (EAL) of 5.9 nm for Ti 2p_3/2_ and 6.2 nm for Sr 3d, which were determined by extrapolating EALs from conventional XPS measurements for epitaxial STO on Ge(001) using a power law for the form 0.060 + $E_{k}^{0.8}$ [1]. We note that the extracted potential profiles are insensitive to the exact values of the EALs over a range of ±0.1 nm from these values.  Experimental measurements made in the same run as those for this STO/Si heterojunction show that the VB maximum in bulk Nb-doped STO(001) is 455.76 eV above the Ti 2p_3/2_ peak energy and 130.26 eV above the Sr 3d_5/2_ peak energy. Subtracting these values from the associated binding energy profiles yields the VB edge profiles shown in Figure S4. The two profiles are in a good mutual agreement with a notable exception of the outermost TiO_2_ plane, where the VB edge shifts upwards by ~0.3 eV.  **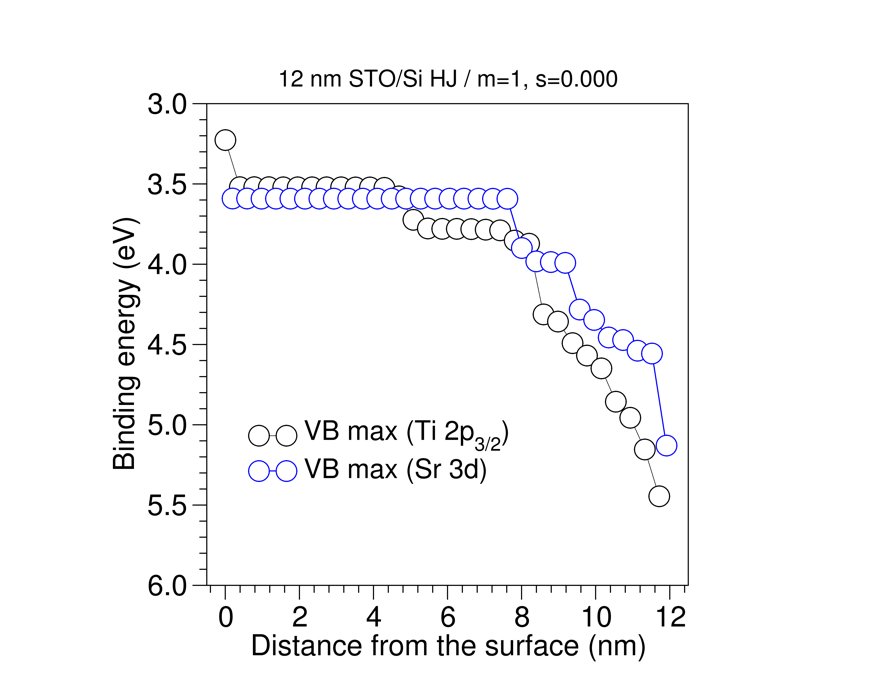**  **Figure S4.** Depth profile of the valence band maximum in a STO (12 nm) / Si heterojunction obtained by fitting the Ti 2p_3/2_ and Sr 3d core-level spectra. The spectra were fitted to extract the dependence of the core-level binding energies on the distance from the STO surface and then shifted by ΔE, where ΔE is the energy difference between the VB maximum and the corresponding core-level peak energy in bulk STO: 455.76 eV for the Ti 2p_3/2_ peak and 130.26 eV for the Sr 3d_5/2_ peak. In both cases, the STO film is assumed to contain 31 TiO_2_ and SrO atomic planes with 100% occupancy of the Ti and Sr sites, respectively.  **Angular dependence.**  HAXPES spectra can be obtained by performing measurements at various take-off angles (θ). To assess their effect on reconstructed band edge profile, we, first, used Eq. 2 and values ΔE_k_ derived by fitting the experimental Ti 2p_3/2_ line shape (θ=85°) to generate line shapes that would have been obtained at lower θ. We selected θ = 48.6°, 30°, and 14.5° so that λ_eff_=λsin(θ) differs from λ corresponding normal incidence by the factors of 0.75, 0.5, 0.25, respectively (Fig. S5(a)).   \| *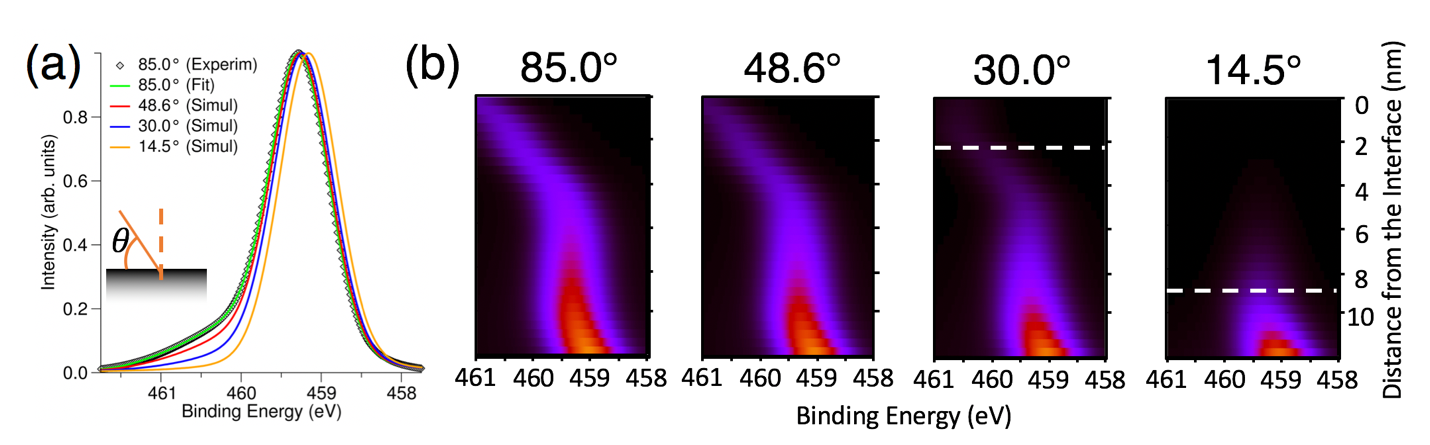* \| \| --- \| \| **Figure S5.** (a) Ti 2p_3/2_ spectrum measured at θ=85° and fitted; the line shapes for other θ are reconstructed from the binding energy profile obtained for θ=85° and shown in Fig. 3(b) (smoothness parameters *m*=1 and *p*=0.256). (b) Families of spatially resolved Ti 2p_3/2_ spectra simulated for selected θ (85°, 48.6°, 30.0°, and 14.5°). Zero on the vertical axes corresponds to the location of the STO/Si interface. The intensity (high = bright, low = dark) decreases with increasing distance from the surface and with decreasing θ. Dashed lines mark the depths at which the intensity become negligible. Adapted from Fig. S11 and S12 in Ref [12]. \|   As θ is reduced, the high binding energy shoulder, which is due to photoemission from the deepest STO layers, decreases in intensity and the portion of the spectrum peaking between 459.0 and 459.5 eV shifts to the lower binding energy. These changes are observed experimentally in STO/Si heterostructures [12]; they are attributed to decreasing sensitivity to the deeper part of the film, where the bands bend downward, with decreasing θ.  Fitting these simulated line shapes generates new sets of binding energy profiles ΔE_k_(θ) shown in Fig. S5(b); the maximums of I_k_(E) associated with individuals planes are shown in Fig. S6. For θ=48.6°, the binding energy profile matches the profile obtained at θ=85°, indicating that λ_eff_ is sufficiently large to probe the entire 12 nm STO film. In contrast, for θ=30°, the cost function W_1_ is sensitive to variations of ΔE_k_(θ) only up to k=25. Deeper TiO_2_ planes provide negligible contribution to the spectrum, which leaves ΔE_k_(θ=30°) undefined for k>25. For θ=14.5°, W_1_ is sensitive only to the contributions from eight near-surface planes.  We note that ΔE_k_(θ) derived for all θ match each other almost exactly in the regions where they are defined as they should since the model spectra I(θ) were generated using one and the same potential profile. |
| ** |
| **Figure S6.** Ti 2p_3/2_ binding energy profiles reconstructed from the experimentally obtained HAXPES line shape measured at the take-off angle θ=85.0° (inset) shown with squares and from line shapes simulated for the lower take-off angles (solid lines) as described in the main text. See also Figure S5. Arrows indicate distances from the surface at which the contributions from deeper TiO_2_ planes become negligible and do not affect the quality of the fit. Accordingly, the binding energies at these planes (dotted lines) are undefined. Adapted from Fig. S11 in Ref [12]. |

| ** |
| --- |
| ** |
| ** |
| **Figure S7.** Fitting of the Si 2p spectrum for the STO (12 nm)/Si heterojunction. (a,b) The dependence of the cost function W_1_ on the number of Si planes for which the binding energies were explicitly optimized. Applying the smoothness correction (m=0) with identical weights (w_k_=1) for all planes k and increasing the magnitude of the smoothness parameter (p) facilitates discrimination between band-bending up (a) and band-bending down (b). (c,d) Experimental and fitted spectra for the upward (c) and downward (d) band-bending. Arrows indicate the regions of largest deviation between the experimental data and fits for p=0.032. (e,f) Residuals for upward (e) and downward (f) band-bending for p=0.032 suggest that the former results in better agreement with experiment than the latter, as discussed in the main text. |

| ** |
| --- |
| **Figure S8.** Fitting of the Ti 2p_3/2_ spectrum for the STO(12 nm)/Si heterojunction. (a) The dependence of cost function W_1_ on the number of TiO_2_ planes used to fit the Ti 2p_3/2_ spectrum (measured at θ=85°). The film thickness corresponds to 31 TiO_2_ planes (indicated with the dashed line). Fitting this spectrum with a smaller number to planes results in a rapidly increasing magnitude of W_1_ that follows an exponentially increasing trend. Conversely, fitting the spectrum with a larger number of planes results in a linearly decreasing W_1_. The same trends are observed with (p=0.001, m=1) and without (p=0.000) smoothness correction. (b) Binding energy profiles across the STO film reconstructed using several assumed film thicknesses (N): from N=27 to N=35 TiO_2_ planes (p=0.001, m=1). The first plane corresponds to the surface, the last plane corresponds to the STO/Si interface. Note that the binding energy profiles in the vicinity of the surface and the potential drops across the film are nearly identical for all considered N. The effect of increasing N amounts to a wider region of the flat binding energy profile where contributions due to additional planes are linearly dependent. |

[1] S. A. Chambers, Y. Du, *J. Vac. Sci. Technol. A*, in press (2020).
